# High prevalence of hearing loss in urban Aboriginal infants: the *Djaalinj Waakinj* cohort study

**DOI:** 10.5694/mja2.51534

**Published:** 2022-05-14

**Authors:** Tamara Veselinović, Sharon A Weeks, Valerie M Swift, Deborah Lehmann, Christopher G Brennan‐Jones

**Affiliations:** ^1^ Telethon Kids Institute Perth WA; ^2^ The University of Western Australia Perth WA; ^3^ Curtin University Perth WA

**Keywords:** Hearing disorders, Infectious diseases, Child health

Otitis media and associated hearing loss are highly prevalent in Indigenous Australian children.^1^ Most relevant prevalence studies have been undertaken in rural and remote regions, although most Indigenous children live in urban areas.^2^ One prospective cohort study found that hearing loss was frequent in young Aboriginal children in a semi‐arid zone of Western Australia,^3^ but its prevalence in Aboriginal infants in urban areas has not been investigated.^4^ We therefore estimated the prevalence of hearing loss in Aboriginal infants in Perth, Western Australia, enrolled in the *Djaalinj Waakinj* cohort study (2017–2021),^5^ and examined the association between otitis media and hearing responses.

The *Djaalinj Waakinj* methodology has been described in detail elsewhere.^5^ Infants underwent routine ear health screenings in their homes at 2–4, 6–8, and 12–18 months of age. A formal hearing assessment was conducted at 9–12 months of age, using free‐field visual reinforcement audiometry in a sound‐treated room to determine hearing responses at 500, 1000, 2000, and 4000 Hz.^6^ The mean hearing response for the more sensitive ear was classified as normal (25 dB), mild (26–40 dB), moderate (41–60 dB) and severe hearing loss (61 dB or more).^7^

The presence of middle ear effusion was assessed using tympanometry. Findings were classified by an audiologist: type A tympanograms were deemed to indicate normal middle ear function (no otitis media), type B tympanograms probable otitis media, and type C tympanograms probable Eustachian tube dysfunction. The *Djaalinj Waakinj* study was approved by the Western Australian Aboriginal Human Ethics Committee (WAAHEC #759) and the Child and Adolescent Health Services Human Ethics Research Committee (CAHS HREC #12).

Sixty‐seven of the 125 enrolled infants completed formal hearing assessments (mean age, 12.1 months; standard deviation, 2.3 months; range, 9–21 months); 41 were boys (61%). Twenty‐one of the 67 infants (31%) had normal hearing, 46 (69%) had some degree of hearing loss (mean response, 36.0 dB; 95% confidence interval [CI], 33.3–38.7 dB), including 22 with mild (33%) and 24 with moderate hearing loss (36%). Mean hearing responses were poorer for the 35 infants with abnormal tympanograms at the time of hearing assessment (40.7 dB; 95% CI, 37.0–44.9 dB) than for the 30 infants with normal tympanograms (30.9 dB; 95% CI, 27.8–33.6 dB) (Box).

Box 1 Mean frequency‐specific hearing responses (with 95% confidence intervals) for Aboriginal infants (about twelve months of age), by tympanogram type*

**Figure:**
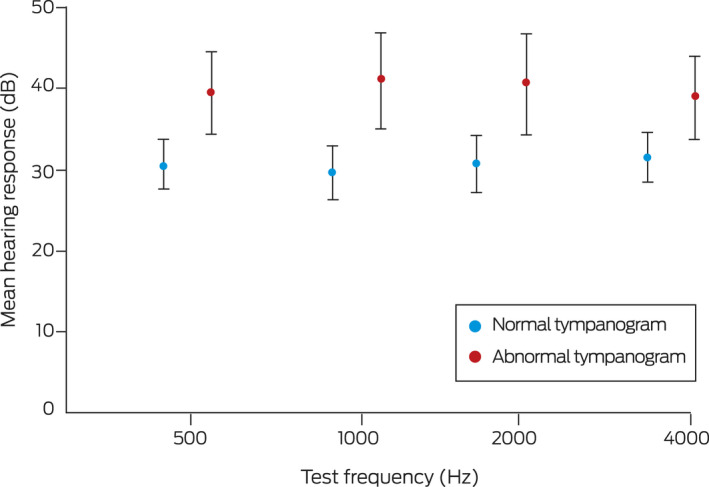
* Normal: type A tympanogram (30 infants); abnormal: type B (30 infants) or type C (five infants). Tympanograms were not available for two infants. Visual reinforcement audiometry was performed with GSI 61 (Grason–Stadler), Equinox (Interacoustics), and Avant (MedRx) audiometers, using pure tone, warble, or filtered narrowband noise stimuli through loudspeakers one metre from the child’s ear at an angle of 90°. Tympanometry was conducted using either Titan Middle Ear Analyser (Interacoustics) or GSI 39 or MI 44 tympanometers (Maico).

Our finding that 69% of urban Aboriginal infants in our study had hearing loss at about twelve months of age is comparable with that of a similar study in Kalgoorlie (65%).^3^ Hearing assessments in infants are based on minimum response levels rather than hearing thresholds,^6^ which probably explains the apparent mild hearing loss in infants with normal tympanograms. The mean hearing response in these children was nevertheless about 10 dB better than in infants with abnormal tympanograms.

The mean hearing response in infants with abnormal tympanograms was 40.7 dB, a level at which they would not hear normal voices clearly, with implications for later speech, language, and behavioural development.^8^ Our findings support early monitoring of otitis media and hearing loss in Indigenous children, with prompt referral for audiological assessment as recommended by Australian otitis media guidelines.^1^

## Open access

Open access publishing facilitated by The University of Western Australia, as part of the Wiley ‐ The University of Western Australia agreement via the Council of Australian University Librarians.

## Competing interests

No relevant disclosures.
